# Prenatal diagnosis of a severe form of frontonasal dysplasia with severe limb anomalies, hydrocephaly, a hypoplastic corpus callosum, and a ventricular septal defect using 3D ultrasound: a case report and literature review

**DOI:** 10.1186/s12884-024-06619-4

**Published:** 2024-06-10

**Authors:** Cuixia Guo, Tiejuan Zhang, Ying Ma, Song Yue, Lijuan Sun

**Affiliations:** 1 Department of Ultrasound, Beijing Obstetrics and Gynecology Hospital, Capital Medical University. Beijing Maternal and Child Health Care Hospital, Beijing, China; 2 Department of Obstetrics, Beijing Obstetrics and Gynecology Hospital, Capital Medical University. Beijing Maternal and Child Health Care Hospital, Beijing, China

**Keywords:** Frontonasal dysplasia, Prenatal diagnosis, 3D ultrasound, Genetics

## Abstract

**Background:**

Frontonasal dysplasia (FND) is a rare congenital anomaly resulting from the underdevelopment of the frontonasal process, and it can be syndromic or nonsyndromic. The typical features of FND include a deformed nose and ocular hypertelorism, which are sometimes associated with cleft lip and/or palate. Only approximately 10 cases of prenatally diagnosed nonsyndromic FND have been reported in the past 30 years.

**Case presentation:**

A 33-year-old woman (G2P1) was referred to our center at 20 gestational weeks for bilateral hydrocephaly. We detected typical features of FND, including severe hypertelorism, median nasal bifidity, a minor cleft lip, and multiple limb anomalies using three-dimensional (3D) ultrasound. A hypoplastic corpus callosum, unilateral microtia, and a ventricular septal defect were also detected. Genetic testing, including karyotype analysis, copy number variation (CNV) analysis, trio-whole exome sequencing (trio-WES), and trio-whole-gene sequencing (trio-WGS), was performed; however, we did not find any de novo gene variants in the fetus as compared to the parents. Postmortem examination confirmed the prenatal diagnosis of FND.

**Conclusion:**

The present case expands the wide phenotypic spectrum of prenatal FND patients. 3D ultrasound is a useful tool for detecting facial and limb deformities.

## Background

Frontonasal dysplasia (FND) is a rare congenital malformation also known as median cleft face syndrome, frontonasal syndrome, or frontonasal dysostosis [1]. For the diagnosis of FND, at least two of the following characteristics must be present: ocular hypertelorism, a broad nasal bridge and/or a bifid nasal tip, a widow’s peak or V-shaped hair line at the forehead, and median facial cleft affecting the nose alone or both the nose and upper lip and, sometimes, the palate or anterior cranium bifidum occultum [2, 3]. The most recognized feature is a deformed nose, which can range from a mild, broad nasal bridge to a severe bifid nasal tip [4].

The severity of the FND phenotype varies considerably from mild to severe, and FND can present as nonsyndromic or syndromic entities that are diverse and genetically heterogeneous. The associated anomalies include ocular and auricular anomalies; central nervous system (CNS) abnormalities; and cardiovascular, urogenital, and limb anomalies [5]. Currently, no consensus has been reached for FND classification. Most cases of FND are sporadic, and only a few patterns have been identified molecular bases.

FND is usually diagnosed clinically by its typical features at birth. Due to its low incidence, the manifestations of the FND spectrum are not well recognized in prenatal diagnosis centers. To date, only 9 fetuses with nonsyndromic FND have been prenatally diagnosed. Here, we described a fetus with typical features of FND, including severe limb anomalies, CNS abnormalities, and cardiac defects detected using prenatal three-dimensional (3D) ultrasound. We also reviewed the relevant literature.

## Case presentation

A 33-year-old pregnant woman, gravida 2, para 1, was referred to our center for bilateral hydrocephalus at 20 gestational weeks. She gave birth to a boy by cesarean section in 2012, and the boy was healthy and without physical deformities. The mother and father were phenotypically normal and denied a family history of genetic malformations. The pregnancy was uneventful without pregnancy complications or exposure to known teratogens.

**Fig. 1:**
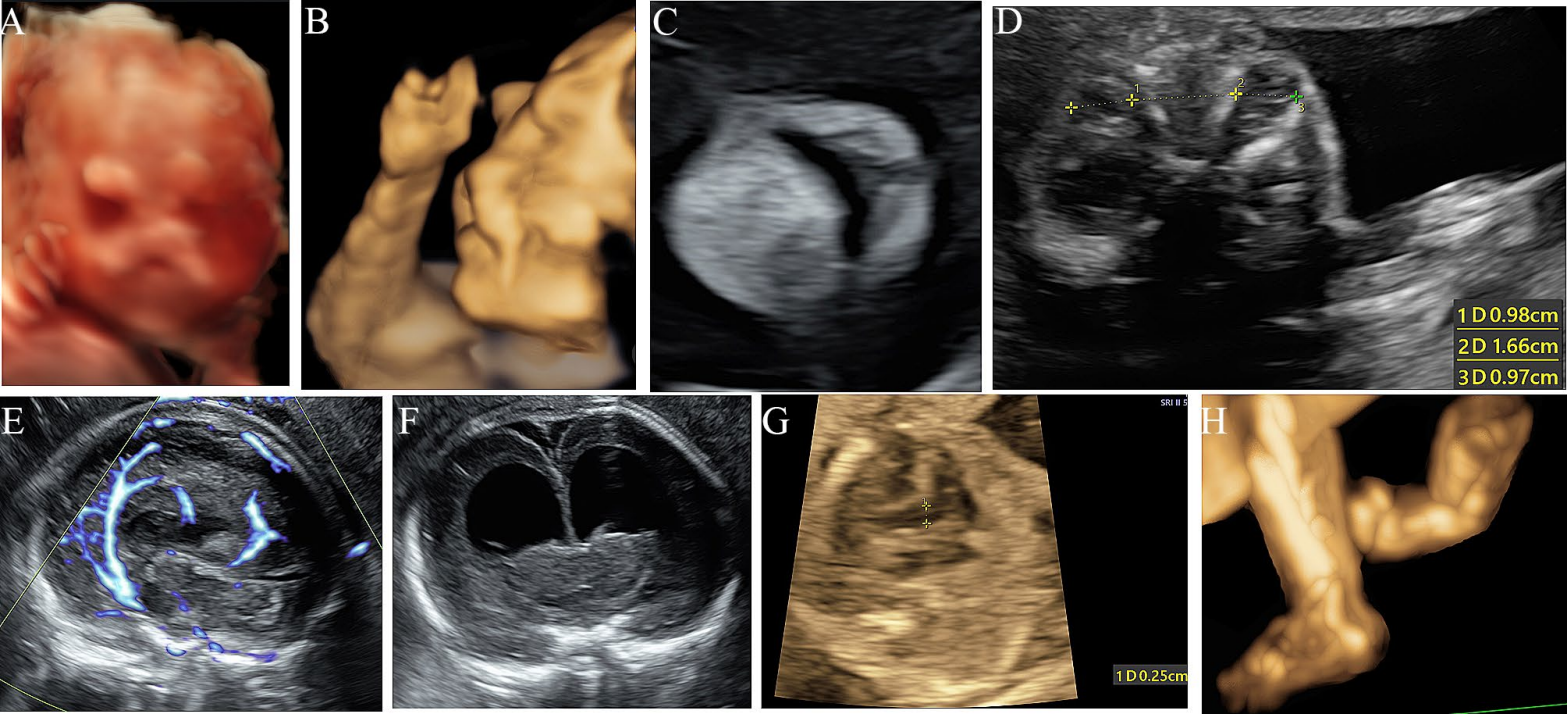
(**a**) 3D surface rendering showing facial dysmorphism, including a broad nasal root without a nasal tip and divided nostrils resembling two eyes. (**b**) 3D ultrasound showing an abnormal profile and a deformed right hand. (**c**) The coronal plane of the face showing a minor median upper lip cleft measuring 1.5 mm. (**d**) The axial plane of the eyes showing ocular hypertelorism, with an interorbital diameter of 16.6 mm and orbital diameters of 9.8 mm and 9.7 mm. (**e**) The abnormally shaped pericallosal artery with high-quality slow flow in the midsagittal plane of the brain demonstrating hypoplasia of the corpus callosum. (**f**) The coronal plane of the brain showing bilateral hydrocephalus with round anterior horns. (**g**) Note a perimembranous ventricular septal defect measuring 2.5 mm. (**h**) 3D ultrasound showing that the left club foot, and the left lower limb were shorter than the right limb

A detailed ultrasonographic examination was performed using a Voluson E10 scanner with a two-/three-dimensional (2D/3D) transducer. Facial dysmorphism, limb anomalies, CNS abnormalities, and cardiac anomalies were detected (Fig. 1). The facial anomalies included ocular hypertelorism with an outer orbital distance of 36.1 mm and an inner orbital distance of 16.6 mm, median nasal bifidity without a nasal tip, and a minor cleft lip measuring 1.5 mm. The limb malformations were severe and included amelia of the left upper limb, syndactyly of the right hand and left foot, hypoplasia of the left tibia and fibula, and a left club foot. Bilateral hydrocephalus with left and right ventricles measuring 18.8 mm and 18.3 mm, respectively, hypoplasia of the corpus callosum, a perimembranous ventricular septal defect (VSD) measuring 2.5 mm, unilateral microtia, and a single umbilical artery were also detected; therefore, FND was diagnosed prenatally.

**Fig. 2:**
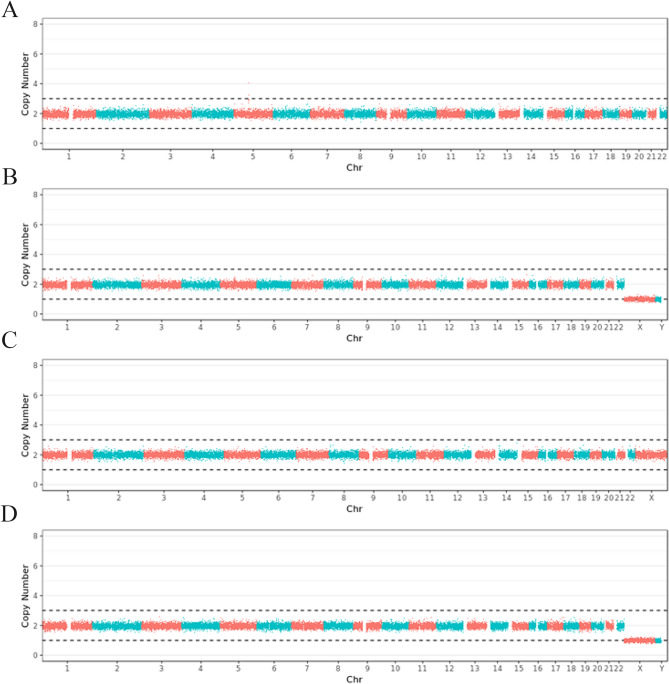
(a-d) The normal whole genome maps of the fetus, the father, the mother, and the brother

Amniotic fluid was collected by amniocentesis at 20 gestational weeks. Karyotype analysis was subsequently performed. Moreover, peripheral blood samples from the couple and their first child were obtained for copy number variation (CNV) analysis and trio-whole exome sequencing (trio-WES). The karyotype of the fetus was 46,XY. The CNV and trio-WES results were negative. Then, trio-whole-gene sequencing (trio-WGS) was conducted, and no de novo gene variants were found in the fetus as compared to the parents (Fig. 2).

**Fig. 3:**
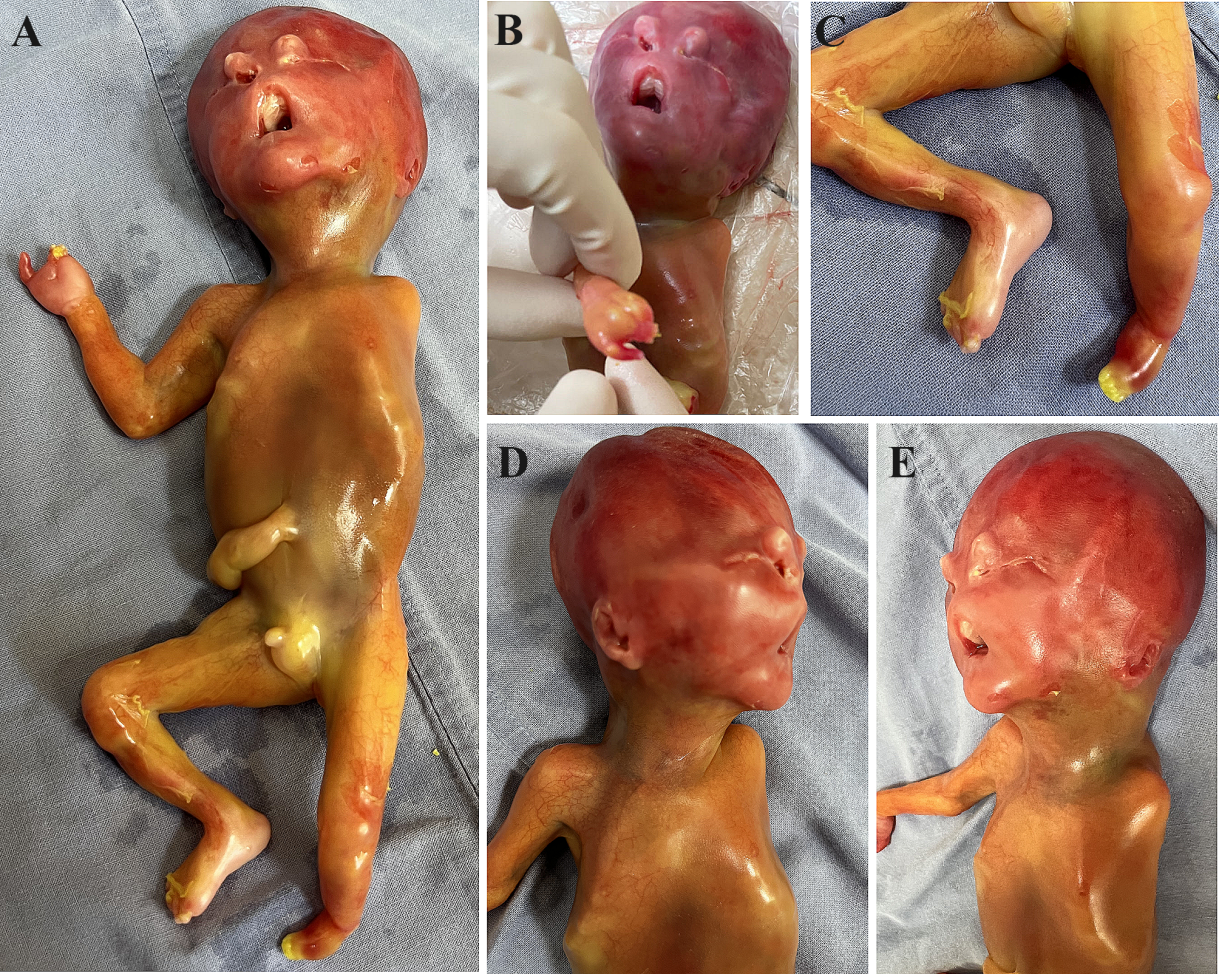
(**a**) A picture of the fetus after induced delivery showing multiple malformations of the face and limbs. (**b**) A picture of the right hand showing syndactyly of four fingers. (**c**) A picture of the lower limbs showing that the left lower limb was hypoplastic with a club foot and syndactyly; note that the left leg was shorter than the right leg. (**d**) The right profile showing a normal right ear. (**e**) The left profile showing left low-set microtia

The parents chose to terminate the pregnancy. Postmortem examination revealed typical FND features and severe limb anomalies (Fig. 3). The two nostrils were separated and at the same level as the eyes, and there was severe ocular hypertelorism. The fetus had syndactyly of the right hand and left foot, and the left arm was absent. The left club foot was small, and the left lower limb was shorter than the right lower limb. Moreover, unilateral low-set microtia was also detected.

The characteristics of prenatally diagnosed nonsyndromic FND cases and the present case are summarized in Table 1. There were 9 prenatally diagnosed cases in total. All prenatally diagnosed patients had hypertelorism, 8 patients had deformed noses, 8 patients had cleft lips/palates, 6 patients had hypoplasia of other craniofacial bones, and 2 patients had abnormal ears. CNS malformations, including agenesis/hypoplasia of the corpus callosum, cephalocele, hydrocephalus, heterotopia, and hemimegalencephaly, were the most commonly observed characteristics. Limb anomalies and heart diseases were not mentioned in these cases. Karyotype analysis was performed for all the patients, CNV analysis was performed for 3 patients, FND-related genes were analyzed for 4 patients, and the results were all negative. The diagnostic criteria for syndromic FND and the facial, limb, and CNS characteristics of these patients are summarized in Table 2.

**Table 1:** The characteristics of prenatally diagnosed nonsyndromic FND patients and the present patient

Case		GA at diagnosis	Sonographic findings	Supplementary information from MRI/postmortem/clinical examinations	Outcome	Genetic test result
1	Recio-Rodríguez, Manuel 2014 [6]	19	Hypertelorism, broad nasal base, midline nasal cleft	Hypoplasia of the nasal bone and olfactory tract, absent olfactory bulb, subependymal heterotopias	Live-born infant, dysmorphic features, NND	Karyotype (46, XY)
2	Shipp TD, 2002 [7]	19.5	Hypertelorism, broad flattened nose, unilateral complete cleft lip/palate, displacement of the frontal bone with herniation	Pseudonasal cystic mass	Live-born infant, delayed development	Karyotype (46, XX)
3	Lourenço C 2021 [8]	20	Hypertelorism, broad nasal root, median nose cleft with bifid nostrils, right preauricular and left submandibular tags	Cranium bifidum occultum, cleft palate, micrognathia, low-set ears, cutaneous tags, widow’s peak, micropenis	TOP	Karyotype/CGH array (-), ALX-related /EFNB1 genes (-)
4	Martinelli P 2002 [9]	22	Severe hypertelorism, absent nasal tip, cleft lip, anterior cephalocele, ACC, hemimegalencephaly	Agenesis of the ethmoid and a hypoplasic sphenoidal body	TOP	Karyotype (46, XY)
5	Virupakshaiah, Akash 2023 [10]	25	Twin B with ACC	Mild hypertelorism, depression of the midface, a midline cleft lip, an alveolar cleft, cephalocele, morning glory disc anomalies	Live-born infant, developmental delay	CMA (-) WES (-)
6	Esmer AC 2013 [11]	27	Hypertelorism, separated nostrils, two facial clefts, frontal encephalocele	Asymmetrical eyes, short palpebral fissures	TOP	Karyotype (46, XY), ALX1 gene (-)
7	Johnstone E 2008 [12]	28	Hypertelorism, wide and flat nose, absent nasal bone, mild micrognathia	Widow’s peak	Live-born infant, NND	Karyotype (46, XX)
8	Chervenak FA 1984 [13]	31	Hypertelorism, a cleft lip, severe hydrocephalus, macrocephaly	Flat nose, mild hypoplastic mandible, stenosis of the aqueduct of Sylvius, hypoplasia of cerebellum	Stillborn	Karyotype (46, XX)
9	John L. Franttarelli 1996 [14]	21	Twin B had hypertelorism, an abnormal nasal structure, a wide midface, occipitoparietal encephalocele	Cleft palate, cranium bifidum occultum, hypoplastic maxilla, simplified ears, partial ACC	Preterm labor with CS	Normal chromosomes
Present case		20	Hypertelorism, nasal bifidity, cleft lip, limb malformations, hydrocephalus, hypoplasia of corpus callosum, and a VSD	Unilateral low-set microtia	TOP	Karyotype (46, XY) CNVs (-) WGS (-)

**Table 2:** Characteristic features of syndromic FND and differential diagnosis among the entities

Syndromic FND	Diagnostic features	Facial features	Limb features	CNS features	Inheritance
Craniofrontonasal dysplasia [15, 16]	FND + craniosynostosis	Hypertelorism, broad nasal root, bifid nose, brachycephaly, frontal bossing, cleft lip and/or palate, facial asymmetry	Grooved nails, broad thumbs, polydactyly, syndactyly, clinodactyly of the fifth finger, asymmetry of the lower limbs	Craniosynostosis, hypoplasia or agenesis of the corpus callosum	X-linked?
Pai syndrome [17, 18]	FND + facial polyps	Cleft lip/palate, bifid nose, cutaneous polyps of the nasal mucosa and face		Pericallosal lipoma, ACC/pACC	Unknown
Oculoauriculofrontonasal dysplasia [19]	FND + oculoauriculovertebral spectrum	Widely spaced eyes, broad nose, mandibular hypoplasia, preauricular tags, facial cleft, ocular dermoids, eyelid colobomata			Unknown
Frontofacionasal dysplasia [20]	FND + cranial and ophthalmic deformities	Defects of the alae nasi and blepharophimosis, lagophthalmos, and S-shaped palpebral fissures, epibulbar dermoids and colobomata of the iris or optic disk		Intracranial lipomata, encephalocele	AR
Acro-fronto-facio-nasal dysostosis [21]	FND + skeletal anomalies, hypospadias (type II)	Hypertelorism, broad notched nasal tip, cleft lip/palate, wide forehead, long philtrum, eye and ear anomalies	Camptobrachypolysyndactyly, polysyndactyly, syndactyly, broad thumbs, fibular hypoplasia	Frontal encephalocele, anomalies of the cortical gyration	AR
Oral-facial-digital syndromes [22, 23]	FND + oral cavity + digit anomalies	Widely spaced eyes, telecanthus, hypoplasia of the alae nasi, median cleft lip and palate, micrognathia, lobulated/bifid tongue, tongue nodules, hypodontia	Brachydactyly, syndactyly, clinodactyly, polydactyly	Intracerebral cysts, ACC, cerebellar abnormalities	X-linked (OFDI) AR (other OFD subtypes)
Acrofacial dysostosis syndromes [24, 25]	Mandibulofacial dysostosis + limb anomalies	Micrognathia, cleft lip and palate, prominent nasal bridge	Phocomelia, oligodactyly, limb deficiency, absent fibulae and tibiae		AD and AR
Acromelic FND [26–28]	FND + brain abnormalities + limb defects	Severe hypertelorism, severe nasal deformities, flat nasal bridge, median cleft nose, cranium bifidum, cleft lip/palate	Polydactyly, tibial hypoplasia, club feet	Encephalocele, callosal dysgenesis, DWM, hydrocephalus	AD
Acrocallosal syndrome [29, 30]	ACC + digital abnormalities, macrocephaly	Broad nasal bridge, normal nasal tip, increased intercanthal distance, high-arched palate, cleft lip/palate, micro/retrognathia	Polysyndactyly, polydactyly	Hypoplastic/absent corpus callosum	AR
Greig Cephalopolysyndactyly [31]	Mild FND + macrocephaly + polysyndactyly	Hypertelorism, broad nasal root, normal nasal tip, frontal bossing	Polydactyly, syndactyly, broad thumbs	Macrocephaly	AD

## Discussion and conclusions

Using 3D ultrasound, we diagnosed a typical fetus with FND, and the coexisting anomalies were severe and rare. The features in the present case were similar to those in acromelic FND, which suggested that they were of the same entity; however, no candidate genes were detected by trio-WES/WGS in our case, which may be a novel unrecognized subtype of FND. The present case emphasizes the wide spectrum of phenotypic variability and genetic heterogeneity of FND. 3D ultrasound plays an important role in demonstrating facial features and deformed limbs in utero.

FND was first described by Sedano et al. in 1970 [2] and likely results from interference in the normal embryological development of the face. FND patients exhibit a wide range of phenotypic features involving the frontonasal process, especially the eyes, nose, and forehead [8, 32]. The persistence of the frontonasal process prevents the orbits from reaching their normal position, which results in ocular hypertelorism and nasal deformities. Most cases of FND are sporadic, and they have been reported as syndromic or nonsyndromic; there is phenotypic overlap between these entities. As the mode of inheritance is still unclear for most patients, the diagnosis is usually based on typical clinical manifestations. Most prenatal nonsyndromic cases lacked positive genetic results, as listed in Table 1. Among syndromic FND patients, some were reported to have AR/AD/X-linked inheritance. The present case involved severe hypertelorism and a prominent nasal bifidity, met the diagnostic criteria for FND, and was differentiated from several syndromic FND, as listed in Table 2.

Reportedly, associated anomalies include microphthalmia, cleft lip/palate, ear tags, facial/intracranial lipoma, hydrocephalus, callosal agenesis, encephalocele, midline cysts, posterior fossa anomalies, and limb and cardiac anomalies [8, 33]. According to the associations, some entities present as rarer syndromes (Table 2). Acromelic FND is characterized by a combination of severe FND, CNS anomalies, and limb defects [28]. It is an autosomal dominant disorder caused by a mutation in the ZSWIM6 gene, although parental mosaic mutations have been reported [34]. Phenotypic overlap in limb and CNS anomalies between the present case and Acromelic FND cases suggested that they were probably the same entity. However, amelia of the upper limb has not been reported in Acromelic FND patients, and no mutations in the ZSWIM6 gene were found in the present patient. Because parental mosaic mutations have been reported in Acromelic FND cases, the diagnosis of Acromelic FND could not be completely excluded.

As shown in Table 2, limb anomalies occur in many syndromic FND patients. Syndactyly and polydactyly are the most common characteristics of these entities. We reviewed the literature, and only one case of FND with amputation of an upper limb in a male patient was reported [35]. The present case was the first case of amelia of an upper limb diagnosed prenatally. Moreover, the fetus had different types of coexisting limb anomalies, which was uncommon within the phenotypic spectrum of FND. Concerning CNS anomalies, agenesis of the corpus callosum (ACC), encephalocele, and lipoma of the corpus callosum are relatively commonly associated with FND [33]. However, hydrocephalus is relatively rare; thus far, only one FND patient with hydrocephalus at 31 gestational weeks has been described [13]. In the present case, the fetus presented with bilateral severe hydrocephalus and hypoplasia of the corpus callosum as early as 20 weeks gestation. Additionally, congenital heart defects, including ventricular/atrial septal defects and hypoplasia of the aortic arch, have been reported in cases of postnatally diagnosed syndromic FND [36]. However, no cardiac defects have been reported in fetuses with FND. This case expanded the prenatal phenotypic spectrum of FND.

The present case should be distinguished from the amniotic band sequence (ABS), which may result from ruptured amniotic membranes wrapping or adhering to the fetus and causing multiple deformities. ABS has a wide spectrum of clinical manifestations involving craniofacial clefts, body wall defects, and limb reductions [37]. The limb deformities in the present case, such as amputation of the limb, syndactyly, edema, and clubfoot, resembled the features of ABS [38]. Additionally, there seemed to be a constriction ring above the left ankle causing hypoplasia of the left lower limb; however, no tissue bands adhering to the fetus were found in utero or during postmortem examination. Surely, it is difficult to distinguish the two entities just by limb anomalies, and FND combined with ABS could not be completely excluded in our patient. The present case shows the phenotypic rarity and diversity of FND.

Most cases of FND are sporadic, and six genes (ALX3, ALX4, ALX1, SIX2, EFNB1, and ZSWIM6) are estimated to account for the minority of FND-associated conditions [3]. Although many patients meet the minimal diagnostic criteria for FND, the associated anomalies do not allow for accurate identification of a distinct syndrome subclass; therefore, the molecular basis is still uncertain for these cases, and the mode of inheritance may be attributed to de novo or mosaic mutations or environmental influences [3]. Although there were differences in limb anomalies between our patient and the patients with acromelic FND and no mutations in the ZSWIM6 gene were detected, the diagnosis of acromelic FND could not be completely excluded.

Our case expanded the prenatal phenotypic spectrum of FND. There is great phenotypic variation and genetic heterogeneity in FND patients. 3D ultrasound is a useful tool for prenatal diagnosis.

## Data Availability

The data are available from the corresponding author upon reasonable request.

## Abbreviations

2D/3D: Two-/three-dimensional
ABS: Amniotic band sequence
ACC: Agenesis of the corpus callosum
AD: Autosomal recessive
AR: Autosomal dominant
CGH: Comparative genomic hybridization
CMA: Chromosomal microarray, TOP: termination of pregnancy
CNS: Central nervous system
CNVs: Copy number variations
CS: Cesarean section, NND: normal neurological development
DWM: Dandy-Walker malformation
FND: Frontonasal dysplasia
pACC: Partial agenesis of the corpus callosum
VSD: Ventricular septal defect
WES: Whole exome sequencing
WGS: whole-gene sequencing
